# N-Type Bismuth Telluride Nanocomposite Materials Optimization for Thermoelectric Generators in Wearable Applications

**DOI:** 10.3390/ma12091529

**Published:** 2019-05-10

**Authors:** Amin Nozariasbmarz, Jerzy S. Krasinski, Daryoosh Vashaee

**Affiliations:** 1 Department of Electrical and Computer Engineering, Monteith Research Center, North Carolina State University, Raleigh, NC 27606, USA; anozari@ncsu.edu; 2 Department of Materials Science and Engineering, North Carolina State University, Raleigh, NC 27695, USA; 3 School of Electrical and Computer Engineering, Helmerich Advanced Technology Research Center, Oklahoma State University, Tulsa, OK 74106, USA; krasins@okstate.edu

**Keywords:** n-type bismuth telluride, thermoelectric materials, nanocomposites, wearable systems, body heat harvesting, power generation

## Abstract

Thermoelectric materials could play a crucial role in the future of wearable electronic devices. They can continuously generate electricity from body heat. For efficient operation in wearable systems, in addition to a high thermoelectric figure of merit, *zT*, the thermoelectric material must have low thermal conductivity and a high Seebeck coefficient. In this study, we successfully synthesized high-performance nanocomposites of n-type Bi_2_Te_2.7_Se_0.3_, optimized especially for body heat harvesting and power generation applications. Different techniques such as dopant optimization, glass inclusion, microwave radiation in a single mode microwave cavity, and sintering conditions were used to optimize the temperature-dependent thermoelectric properties of Bi_2_Te_2.7_Se_0.3_. The effects of these techniques were studied and compared with each other. A room temperature thermal conductivity as low as 0.65 W/mK and high Seebeck coefficient of −297 μV/K were obtained for a wearable application, while maintaining a high thermoelectric figure of merit, *zT*, of 0.87 and an average *zT* of 0.82 over the entire temperature range of 25 °C to 225 °C, which makes the material appropriate for a variety of power generation applications.

## 1. Introduction

One of the most promising technologies for waste heat recovery from environmental sources is based on thermoelectric generators in which the temperature gradient is directly converted to electricity. The conversion efficiency of the thermoelectric materials depends on the thermoelectric dimensionless figure of merit, *zT*, i.e.:

(1) $$zT=\frac{S^{2}\sigma }{\kappa }T$$

where *S* is the Seebeck coefficient in μV/K, σ is the electrical conductivity in S/cm, *κ* is the thermal conductivity (including lattice component *κ_L_*, electronic component *κ_e_*, and bipolar component *κ_bi_*) in W/mK, and *T* is the absolute temperature in *K* [1]. Improving *zT* has always been challenging because *S*, *σ*, and *κ* are interdependent.

The electrical conductivity is defined by *σ = neμ*, where *n*, *e*, and *μ* are the charge carrier concentration, charge (=1.6 × 10^−19^ coulombs) and mobility, respectively [2]. By adding this to Equation (1) and rearranging, we can write:

(2) $$zT=(S^{2}n)\left(\frac{\mu }{\kappa }\right)eT$$

The components in the two parentheses are counter-indicated [3]. According to the Pisarenko relation [4], higher *n* results in lower *S*, while defects reduce both *κ* and *μ*. Therefore, there is always a trade-off among these properties.

While the initial application of thermoelectric materials for power generation was in spacecraft such as Voyager, Cassini, and New Horizons [5], they have recently been used in other applications, such as in vehicles for waste heat recovery, and power generation from body heat in self-powered wearable health sensors [6,7,8,9]. Traditionally, studies have been focused primarily on improving only *zT* of the thermoelectric materials [6,10,11,12,13,14,15,16]. Thus, high *zT* values have been achieved for a variety of materials [17,18,19,20,21]. However, as we will discuss, the individual material properties, namely *κ* and *S*, can play key roles even more than *zT* when the material is used in wearable systems.

A thermoelectric generator (TEG) produces electricity by recovering energy from the (waste) heat of environmental sources. This unique capability makes TEGs a favorable method for energy harvesting from the body. Thermal energy is a continuous energy source from the body, in contrast to mechanical input applied by the user, sunlight, or ambient radio waves, which can be interrupted at times. A self-powered wearable device requires control of the performance of thermoelectric materials and device together [22]. For body wearable TEGs, the performance is limited by the hot side (skin) and cold side (ambient) heat exchangers due to large parasitic thermal resistances [23]. In addition to a high *zT*, the material must have low thermal conductivity. This requirement is mainly due to the skin thermal resistance and the constraints limiting the use of a heat sink in a wearable platform. A larger output voltage at the device level is also required to run the boost converter and the power management unit efficiently. This is fulfilled through materials with high Seebeck coefficients. Therefore, these conditions require the TEG to be designed not only with specific consideration of leg geometries and spacing, but also careful consideration of material properties.

Bismuth telluride (Bi_2_Te_3_) alloys have been developed since the 1960s, and are the most promising thermoelectric materials for low-temperature applications [24]. N-type Bi_2_Te_3_ alloys theoretically suffer from low *zT* due to the restricted number of valleys near the conduction band edge. Recently, researchers have reported *zT* improvements in n-type Bi_2_Te_3_ alloys using zone melting [25], nanostructuring [26,27,28], nano-inclusions [29], texturing [30], and point defect engineering [31]. Although there are a few studies that have shown high *zT* values for n-type Bi_2_Te_3_, these materials are not appropriate for applications such as body heat harvesting due to their non-optimized properties at body temperature. Therefore, optimizing the transport properties of n-type Bi_2_Te_3_ alloys around body (or room) temperature is highly desired.

For body heat harvesting applications, thermoelectric materials require a combination of high *zT*, low *κ*, and high *S* at room temperature [23]. For applications at above 100 °C (i.e., power generation), low *κ* is not determinative; however, high *S* and *zT* are still demanded. To fulfill these requirements, we synthesized optimized nanostructured n-type materials based on Bi_2_Te_2.7_Se_0.3_. We investigated the effect of several parameters, including dopant addition, tellurium vacancies, glass inclusion, sintering time and temperature, microwave processing, and subsequent annealing, primarily to fully optimize the thermoelectric properties of bulk nanostructured n-type Bi_2_Te_2.7_Se_0.3_ materials. N-type materials with a thermal conductivity as low as 0.65 W/mK, Seebeck coefficients as high as −297 μV/K, peak *zT* of 0.87, and average *zT* of 0.82 were achieved. The results show that different parameters can shift maximum *zT* from room temperature to above 100 °C. Materials with excellent properties at room temperature are desired for wearable applications, while those with good features at above 150 °C are useful for high-temperature power generation.

## 2. Experimental Section

Stoichiometric ratios of high purity elements of Bi powder (99.9% purity), Te lump (99.9% purity), and Se powder (99.9% purity) were milled for two hours in a planetary ball mill (Fritsch-P7) using a tungsten carbide bowel and zirconia balls under 950 rpm speed and ball-to-powder ratio of 5:1 to prepare uniform nano-sized Bi_2_Te_3−x_Se_x_ powders. The milling process of all samples was similar.

Additionally, the stoichiometric ratio of high purity elements of Bi powder and Te lump were milled for 15 min in similar conditions and melted in an induction furnace (IF) to prepare homogenized Bi_2_Te_3_ ingots. The process was fulfilled in a graphite die inside a quartz tube under argon atmosphere. The ingots were crushed and milled for 5 h in a planetary ball mill (Fritsch-P6) using a tungsten carbide bowel and balls under 500 rpm and a ball-to-powder ratio of 10:1 to prepare a uniform nano-sized Bi_2_Te_3_ powders. Se of 6 atomic percent (at. %) was subsequently added to the Bi_2_Te_3_ powder and milled for one hour to obtain a uniform powder. To tune the thermal conductivity and Seebeck coefficient, 2.5 at. % glass (Ferro-EG 2964) powder was added to the mixture. The names, compositions, synthesis parameters, room temperature Seebeck coefficient, thermal conductivity, *zT*, and maximum *zT* of the synthesized materials are further listed in Table 1.

The synthesized powders were sintered in a cylindrical graphite die with an inner diameter of 6 mm at different temperatures using spark plasma sintering (SPS), to make the thermoelectric ingots with 6 mm diameter and 14 mm height. The temperature was monitored using a k-type thermocouple placed at the center of the die, close to the center of the sample.

**Figure 1:**
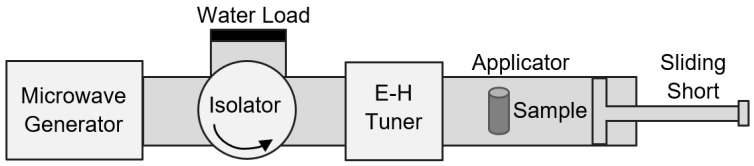
Schematic view of the microwave (MW) set-up. The sample is placed at the center of the waveguide where the electric field is maximum.

The effect of microwave (MW) processing was studied using a rectangular single mode cavity attached to a 2.45 GHz (1 kW) generator. The experimental set up is shown in Figure 1. It contains a magnetron as the source that provides a stable and tunable MW energy [32]. A waveguide was used to direct the electromagnetic wave and restrict the field. An electric and magnetic field (E-H) tuner and a sliding short was used for convenient tuning and load matching during the exposure of the specimen [33,34]. The sample was placed in the center of the waveguide where the electric field is maximum. The material was heated up volumetrically by MW absorption, leading to rapid sintering [35,36].

The electrical resistivity and Seebeck coefficients of the samples were simultaneously measured using the commercially available Ulvac instrument, ZEM-3, from room temperature to 250 °C. The thermal diffusivity (*D*) was measured by a laser flash instrument (Netzsch’s LFA 457 Micro Flash) from room temperature to 250 °C. The specific heat capacity (*C_p_*) was calculated for Bi_2_Se_0.3_Te_2.7_ alloy according to the theoretical data. The thermal conductivity of the samples was derived from the relationship *κ = ρDC_p_*, where *ρ* is the mass density of the samples measured using Archimedes’ principle.

SPS and MW are used in this paper as the abbreviation for spark plasma sintering and microwave, respectively.

## 3. Results and Discussion

### 3.1. Effect of Dopant Addition

In Bi_2_Te_3_ alloy, excess Bi and Te atoms lead to p- and n-type semiconducting behavior, respectively. These excess atoms make anti-site defects by penetrating into the lattice structure [37]. In fact, the doping mechanism in a pure Bi_2_Te_3_ alloy is mainly through anti-site and vacancy defects. The Bi_Te_ anti-site donates one hole, and the Te_Bi_ anti-site provides one electron. Furthermore, Bi vacancy donates 3 holes, and Te vacancy provides 2 electrons [38,39,40,41]. The formation energy of Te_Bi_ anti-site defect is lower than the Te vacancy and Bi_Te_ anti-site, hence, it is the most abundant doping mechanism in Bi_2_Te_3_ alloy [40]. Formation of Te vacancies in n-type Bi_2_Te_3_ is unavoidable, resulting in the presence of the Fermi level deep in the conduction band that reduces the power factor (*S*^2^*σ*) [28]. Addition of Se to Bi_2_Te_3_ can tune the anion vacancies, which results in power factor enhancement [31].

**Figure 2:**
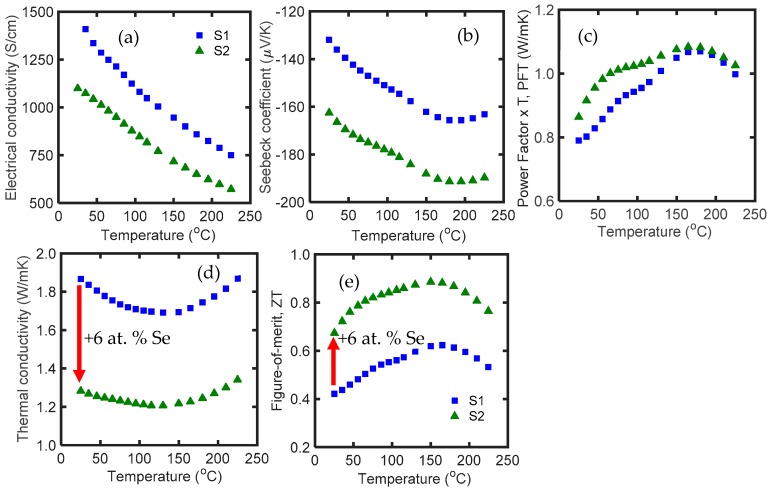
(**a**) Electrical conductivity; (**b**) Seebeck coefficient; (**c**) power factor × temperature; (**d**) thermal conductivity; and (**e**) *zT* of S1 and S2 as a function of temperature in the range of 25 °C to 225 °C. According to Table 1, S1 is a pure Bi_2_Te_3,_ and S2 is Bi_2_Te_3_ with 6 at. % Se.

Figure 2 shows electrical conductivity, Seebeck coefficient, power factor times temperature (PFT), thermal conductivity, and *zT* of S1 and S2 as a function of temperature in the range of 25 °C to 225 °C. The Seebeck coefficient and high electrical conductivity of S1 reveals that it has an excessively high carrier concentration. The negative sign of the Seebeck coefficient is due to the n-type characteristic of Bi_2_Te_3_. Addition of Se dopant to S1 tuned the carrier concentration and improved the power factor up to 13%, and decreased the thermal conductivity by 30%. This resulted in a *zT* of 0.67 at room temperature in S2, which is 60% higher than that of S1. S2 also showed an average *zT* of 0.82 in the temperature range of 25 to 225 °C. The bipolar effect in this alloy occurs at ~150 °C, and addition of selenium dopant does not shift the bipolar effect to a lower temperature.

### 3.2. Optimizing Te Vacancy

**Figure 3:**
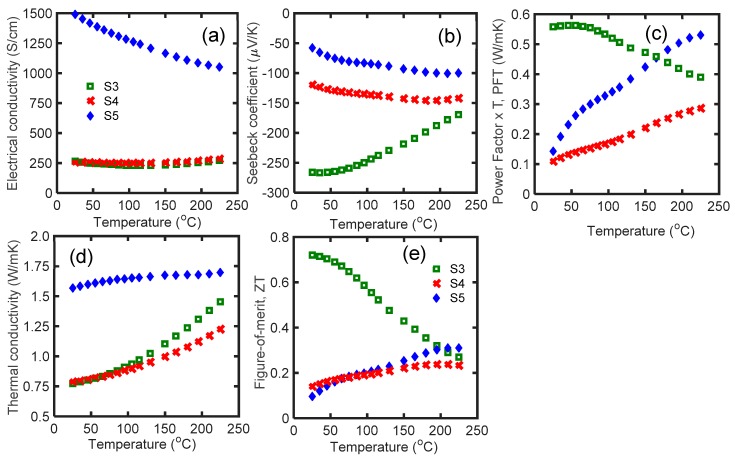
(**a**) Electrical conductivity; (**b**) Seebeck coefficient; (**c**) power factor × temperature; (**d**) thermal conductivity; and (**e**) *zT* of S3, S4, and S5 samples as a function of temperature in the range of 25 °C to 225 °C. The compositions of S3, S4, and S5 are Bi_2.00_Te_2.70_Se_0.3_, Bi_2.04_Te_2.66_Se_0.3_, and Bi_2.15_Te_2.55_Se_0.3_, respectively.

Te vacancy can control the thermoelectric properties of n-type Bi_2_Te_3_ alloys by adjusting the Fermi level and transport properties. According to Figure 3, the reduction of Te content from the stoichiometric ratio (i.e., S4 and S5) led to a dramatic drop in thermoelectric properties. Te vacancies result in highly doped n-type materials. According to the extremely high electrical conductivity and low value of the Seebeck coefficient, S5 is a highly doped material with electrical conductivity six times higher than S3 at room temperature (Figure 3a,b). The electrical conductivity of S4 was identical to S3 in all the temperature ranges (Figure 3a). The low electrical conductivity of S4 is due to the small carrier mobility. S3 had the highest PFT below 150 °C (Figure 3c). Smaller PFT in S4 and S5 is due to the Te vacancies, which move the Fermi level deep in the conduction band [28]. The thermal conductivity of S3 and S4 was similar up to 100 °C, then it differentiated at higher temperatures (Figure 3d). Non-optimized thermoelectric properties of S4 and S5 resulted in very low *zT* in these samples. According to this result, for 6 at. % Se, Bi_2.0_Te_2.7_Se_0.3_ has the optimum Te vacancy concentration. Selenium dopant effectively compensated Te vacancies in S3, and it had a maximum *zT* of 0.72 at room temperature. Further *zT* improvement of S4 and S5 requires optimization of the amount of Se in this alloy.

### 3.3. Effect of Glass Inclusion and Soaking Time

**Figure 4:**
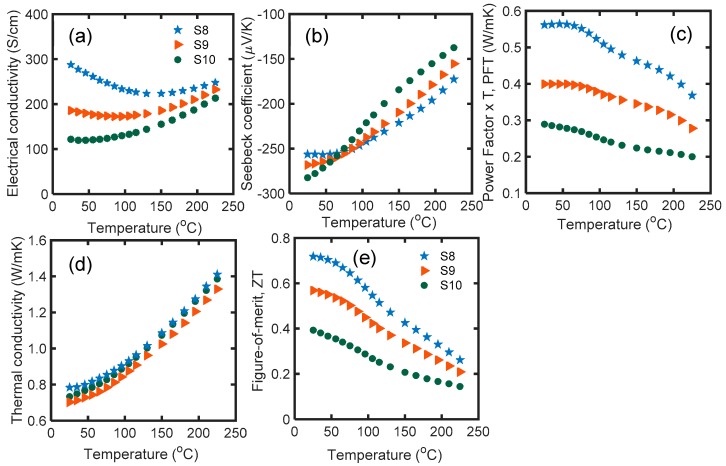
(**a**) Electrical conductivity; (**b**) Seebeck coefficient; (**c**) power factor times temperature (PFT); (**d**) thermal conductivity, and (**e**) *zT* of S6, S7, and S8 samples as a function of temperature in the range of 25 °C to 225 °C. A 2.5 at. % glass inclusion was added to Bi_2.0_Te_2.7_Se_0.3_. S6, S7, and S8 were sintered at 540 °C with 1, 2 and 3 min soaking time, respectively.

Figure 4 shows the thermoelectric properties of S6, S7, and S8 as a function of temperature in the range of 25 °C to 225 °C. The effects of addition of 2.5 at. % glass inclusion into Bi_2.0_Te_2.7_Se_0.3_ along with 1, 2, and 3 min soaking times were studied. All samples were sintered at 540 °C.

The electrical conductivity of the samples reduced with increased soaking time (Figure 4a). S8 had the highest electrical conductivity at room temperature, equal to 287 S/cm, and S10 had the lowest, equal to 121 S/cm. The electrical conductivity of the samples with glass inclusions dropped with soaking time. The variation of electrical conductivity among the samples become small at higher temperatures (>150 °C) due to the dominance of the acoustic phonon scattering mechanism.

Glass inclusion improved the Seebeck coefficient of the samples, and the soaking time had an extra constructive effect on increasing the Seebeck coefficient at room temperature. S8 had the highest Seebeck coefficient, equal to −283 μV/K. The absolute value of the Seebeck coefficient decreased with temperature over the entire temperature range. Over 75 °C, S6 showed the maximum Seebeck coefficient. The glass nano-inclusions provide new barriers for the charge carriers (i.e., electrons). High energy electrons can overcome the barrier energy and pass through the barrier; however, low energy electrons are reflected. This technique increases the mean energy of the electrons with respect to the Fermi energy, and thus increases the overall Seebeck coefficient.

The PFT of all samples decreased with temperature, and the soaking time impaired the PFT of the nanocomposites. S6 had the maximum PFT of 0.56 W/mK at room temperature. Thermal conductivity was monotonically enhanced with temperature in all glass-included samples. S6 had the highest room temperature thermal conductivity, equal to ~0.78 W/mK, and higher soaking times resulted in a reduction of the thermal conductivity up to 10% (Figure 4d).

All *zT*s were maximum near room temperature. The maximum *zT* values of the S6, S7, and S8 samples were 0.72, 0.57 and 0.39, respectively (Figure 4e). The sample with 1 min soaking time had the highest *zT* throughout the temperature range. Although longer soaking time led to an improved Seebeck coefficient and reduced the thermal conductivity by ~10%, it had a dramatic destructive effect on *zT* of n-type nanocomposites of Bi_2.0_Te_2.7_Se_0.3_-2.5% glass samples by up to 46%. Therefore, for specimens with glass inclusions, shorter soaking time is required.

### 3.4. Effect of Microwave Processing with Glass Inclusion

**Figure 5:**
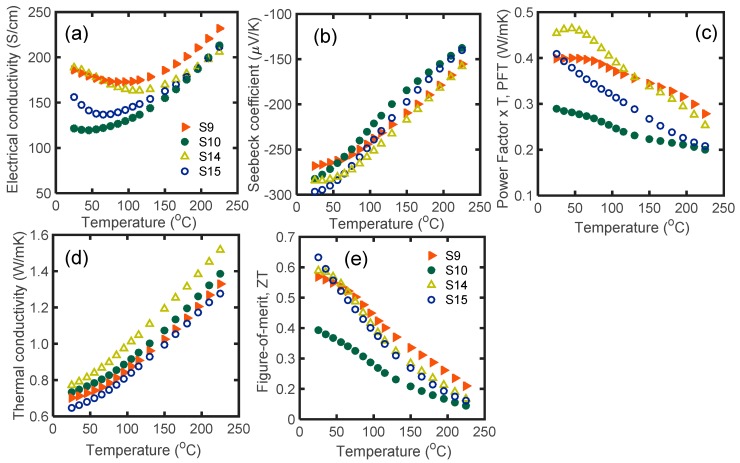
(**a**) Electrical conductivity; (**b**) Seebeck coefficient; (**c**) PFT; (**d**) thermal conductivity; and (**e**) *zT* of S7, S8, S9, and S10 as a function of temperature in the range of 25 °C to 225 °C. According to Table 1, S9 and S10 are the microwave processed forms of S7 and S8, respectively.

Figure 5 depicts the thermoelectric properties of S7, S8, S9, and S10 samples as a function of temperature in the range of 25 °C to 225 °C. S9 and S10 are S7 and S8 after microwave processing, respectively. As mentioned, S7 and S8 are samples with 2.5 at. % glass inclusions, sintered at 540 °C for 2 and 3 min, respectively.

The room temperature electrical conductivity of S7 did not change after microwave radiation (S9), while the room temperature electrical conductivity of S8 was improved by 30% after microwave processing (S10). At higher temperatures, microwave processing resulted in an electrical conductivity drop in S9, while it did not affect S10 (Figure 5a).

The absolute value of the Seebeck coefficients of S9 and S10 improved by 6% and 5%, respectively. S10 had the maximum Seebeck coefficient, equal to −297 μV/K (Figure 5b), which is one of the highest reported values in the literature. The PFT of S7 and S8 increased by 13.7% (S9) and 42% (S10) respectively after microwave processing (Figure 5c). The improved thermoelectric PFT of the microwave processed samples in comparison to SPS samples is also due to the non-linear transport of charge carriers between the decrystallized grains with compositional variation and/or disordered-crystalline regions [42,43,44].

Glass inclusion generally reduces the thermal conductivity of Bi_2.0_Te_2.7_Se_0.3_. The thermal conductivity of all glass-included samples increased monotonically with temperature. According to Figure 5d, the thermal conductivity of S9 (S7 after microwave processing) was increased by 10%. In contrast, the thermal conductivity of S10 (S8 after microwave processing) dropped by 12%. Because S8 initially had poor thermoelectric properties, it is probable that glass inclusion played a constructive role in reducing the thermal conductivity of S10.

The microwave processed samples (S9 and S10) had higher *zT* values compared to their initial SPS samples (S7 and S8). The *zT* of all samples decreased with temperature over the entire temperature range. Figure 5e shows that S10 had the highest *zT*, equal to 0.63. At room temperature, *zT* of S8 increased by 60% after microwave processing, and *zT* of S9 slightly improved after microwave processing. In general, microwave processing enhanced the electrical conductivity, Seebeck coefficient, and *zT* of the glass-included samples, which shows that microwave processing is a reliable technique with which to engineer the thermoelectric properties of materials.

### 3.5. Effect of Initial SPS Temperature

**Figure 6:**
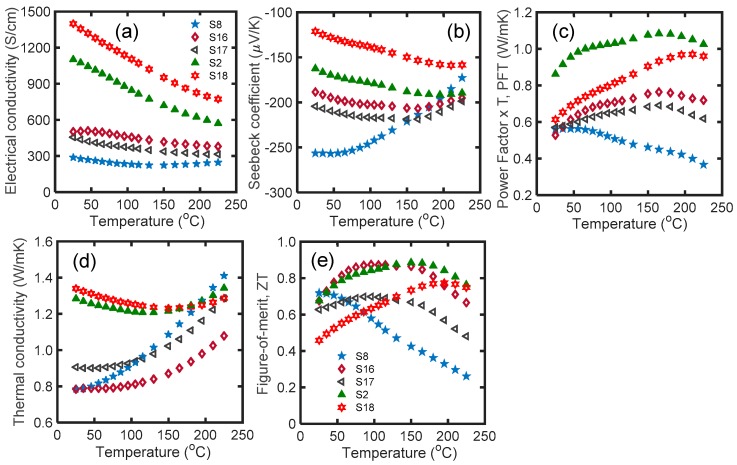
(**a**) Electrical conductivity; (**b**) Seebeck coefficient; (**c**) PFT; (**d**) thermal conductivity; and (**e**) *zT* of S6, S11, S12, S2, and S13 samples as a function of temperature in the range of 25 °C to 225 °C. According to Table 1, the composition and processing of the samples are as follows: S6: Bi_2.0_Te_2.7_Se_0.3_-2.5% glass, SPS at 540 °C for 1 min; S11: Bi_2.0_Te_2.7_Se_0.3_-2.5% glass, SPS at 310 °C for 1 min, microwave processed and post SPS at 450 °C; S12: Bi_2.0_Te_2.7_Se_0.3_-2.5% glass, SPS at 270 °C for 1 min, microwave processed and post SPS at 450 °C; S2: Bi_2_Te_3_-6 at. % Se, SPS at 540 °C for 1 min, microwave processed and post SPS at 450 °C; and S13: Bi_2_Te_3_-6 at. % Se, SPS at 310 °C for 1 min, microwave processed and SPS at 450 °C.

Figure 6 shows (a) electrical conductivity, (b) Seebeck coefficient, (c) PFT, (d) thermal conductivity, and (e) *zT* of S6, S11, S12, S2, and S13 as a function of temperature in the range of 25 °C to 225 °C. S6 and S2 are depicted in this figure for comparison with other samples.

S6, S11, and S12 contained glass inclusions. Comparison of the electrical conductivity of these samples revealed that initial SPS at 310 °C, along with microwave processing, resulted in larger electrical conductivity (Figure 6a). The electrical conductivity of S11 decreased from 501 S/cm at room temperature to 378 S/cm at 225 °C. Similarly, for Bi_2_Te_3_-6 at. % Se samples (S2 and S13), lower initial hot press temperature resulted in higher electrical conductivity throughout the temperature range. The electrical conductivity of S2 and S13 monotonically decreased with temperature. It seems that the carrier concentration in low-temperature SPS samples is higher than others.

S11, S12, S2, and S13 showed lower Seebeck coefficients compared to the previous samples (Figure 6b). Since the Seebeck coefficient is a strong function of the carrier concentration [45], a smaller absolute value of the Seebeck coefficient confirms the higher carrier concentration in all low-temperature SPS samples.

S6, S11, and S12 (all with glass inclusion) had almost similar PFT values, ~0.55 W/mK at room temperature (Figure 6c). The PFT of S11 and S12 increased up to 170 °C, then decreased. S2 and S13 (samples without glass inclusion) had higher PFT values throughout the temperature range. S2 had 20% higher PFT than S13, and its PFT was >1 W/mK over 65 °C.

S11 had similar thermal conductivity to S6 at room temperature, and its thermal conductivity increased by a lower slope at a higher temperature. The variation of thermal conductivity versus temperature for samples with lower initial SPS temperature (i.e., 310 and 270 °C) was less than the samples which were initially sintered at 540 °C. The thermal conductivity of S12 was higher than S11 throughout the temperature range. S2 and S13 had greater room temperature thermal conductivity. It can be concluded that nanocomposite samples with glass inclusions, sintered at either low or high temperature, generally show lower thermal conductivity compared to the nanostructured samples sintered with similar conditions.

S6 had the highest *zT* at room temperature (Figure 6e). For glass included samples, S11 had a higher *zT* over 35 °C, and it had an average *zT* of 0.81 from 25 °C to 225 °C. Among the S6, S11, S12, S2, and S13 samples, S6 had the best properties for wearable application due to its having the highest Seebeck coefficient, lowest thermal conductivity, and highest *zT* at room temperature. Additionally, samples S2 and S11 had *zT* > 0.8 in the broad range of 50–175 °C. These samples are appropriate candidates for power generation applications. For the glass containing samples, initial SPS at lower temperature led to higher electrical conductivity, lower Seebeck coefficient, and similar thermal conductivity over the whole temperature range.

### 3.6. Effect of Annealing

**Figure 7:**
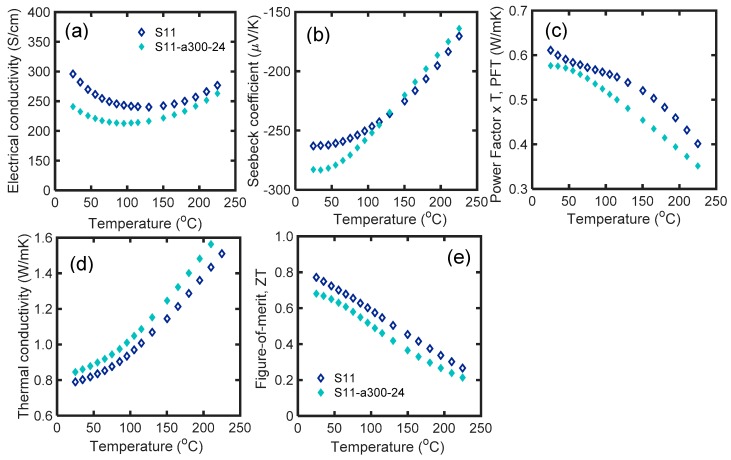
(**a**) Electrical conductivity; (**b**) Seebeck coefficient; (**c**) PFT; (**d**) thermal conductivity; and (**e**) *zT* of S14 and S14-a300-24 samples as a function of temperature in the range of 25 °C to 225 °C. According to Table 1, S14-a300-24 is S14 after annealing at 300 °C for 24 h.

Figure 7 shows the thermoelectric properties of S14 and S14-a300-24 as a function of temperature in the range of 25 °C to 225 °C. The processing conditions are explained in Table 1. The electrical conductivity of S14 decreased throughout the temperature range after 24 h annealing at 300 °C. The room temperature electrical conductivity dropped by 18.5%, while the absolute value of the Seebeck coefficient increased by 8% after annealing. The carrier concentration has dropped probably due to the annealing of the anti-site and vacancies. The PFT of the annealed sample fell over the entire temperature range. The thermal conductivity of the annealed sample increased due to the grain growth at 300 °C. Increased grain size results in lower interaction of phonons with grain boundaries that can improve the *κ_L_*. The *zT* values of both samples monotonically decreased by temperature. The room temperature *zT* of S14 dropped by ~12% after annealing. The variation between the *zT* of S14 and S14-a300-24 was almost constant over the temperature range. It is concluded that, although annealing at 300 °C resulted in higher Seebeck coefficient at room temperature, it increased the thermal conductivity and reduced the *zT*.

## 4. Conclusions

We synthesized high-performance nanocomposites of n-type Bi_2_Te_2.7_Se_0.3_ for body heat harvesting and power generation applications. The effects of several parameters, including dopant addition, tellurium vacancy, glass inclusion, microwave processing, SPS soaking time and temperature, and subsequent annealing were studied to optimize the thermoelectric properties of the synthesized materials. In general, the addition of a Se dopant resulted in both power factor enhancement and thermal conductivity reduction. Tellurium vacancy can drop the power factor of n-type Bi_2_Te_3_ alloys significantly. An optimum amount of selenium dopant is required to compensate for the loss. Glass inclusion reduced the thermal conductivity and improved the Seebeck coefficient of the alloys. However, a longer soaking time decreased the *zT* of the glass-included samples. Microwave processing enhanced the electrical conductivity, Seebeck coefficient, and *zT* of the glass-included samples. It proved to be a reliable method to improve the thermoelectric properties of different materials. Annealing at 300 °C increased the thermal conductivity and reduced the *zT*, while it increased the Seebeck coefficient.

A room temperature thermal conductivity of 0.65 W/mK, Seebeck coefficient of −297 μV/K and *zT* = 0.76 were achieved which fulfill the combined requirements (low thermal conductivity, high Seebeck coefficient and high *zT* at room temperature) for body heat harvesting applications. The optimized materials in this study showed a peak *zT* of 0.87, and an average *zT* of 0.82 over the entire temperature range of 25–225 °C, and a high Seebeck coefficient, above −200 μV/K, which makes them suitable for a variety of power generation applications.

## Figures and Tables

**Table 1:** List of names, compositions, synthesis parameters, room temperature Seebeck coefficients, thermal conductivities, *zT*, and maximum *zT* of the synthesized materials.

Name	Material	1st SPS T (°C)/Soak Time (min) *	MW Process	2nd SPS T (°C)/Soak Time (min)	Room T *S* (μV/K)	Room T *κ* (W/mK)	Room T *zT*	Max *zT*
S1	Bi_2_Te_3_	540/1	Yes	450/1	−132	1.95	0.37	0.57
S2	Bi_2_Te_3_-0.3%Se **	540/1	Yes	450/1	−163	1.28	0.70	0.89
S3	Bi_2_Te_2.7_Se_0.3_	540/2	No	-	−266	0.78	0.72	0.72
S4	Bi_2.04_Te_2.66_Se_0.3_	540/2	No	-	−120	0.79	0.15	0.24
S5	Bi_2.15_Te_2.55_Se_0.3_	540/2	No	-	−58	1.57	0.11	0.33
S6	Bi_2_Te_2.7_Se_0.3_-2.5%Glass	540/1	No	-	−256	0.78	0.72	0.72
S7	Bi_2_Te_2.7_Se_0.3_-2.5%Glass	540/2	No	-	−268	0.70	0.57	0.57
S8	Bi_2_Te_2.7_Se_0.3_-2.5%Glass	540/3	No	-	−283	0.73	0.39	0.39
S9	Bi_2_Te_2.7_Se_0.3_-2.5%Glass	540/2	Yes	450/1	−284	0.77	0.59	0.59
S10	Bi_2_Te_2.7_Se_0.3_-2.5%Glass	540/3	Yes	450/1	−297	0.65	0.63	0.63
S11	Bi_2_Te_2.7_Se_0.3_-2.5%Glass	310/1	Yes	450/1	−188	0.78	0.67	0.87
S12	Bi_2_Te_2.7_Se_0.3_-2.5%Glass	270/1	Yes	450/1	−204	0.91	0.63	0.70
S13	Bi_2_Te_3_-0.3%Se	310/1	Yes	450/1	−121	1.34	0.46	0.77
S14	Bi_2_Te_2.7_Se_0.3_	540/2	Yes	450/1	−263	0.79	0.76	0.76
S15-a300-24	Bi_2_Te_2.7_Se_0.3_ (anneal at 300°C/24h)	540/2	Yes	450/1	−283	0.85	0.68	0.68

* Table abbreviations: SPS: spark plasma sintering, T: temperature, MW: microwave. ** 0.3% Se is equal to 6 at. % Se.
